# Processed ready-to-eat (RTE) foods sold in Yenagoa Nigeria were colonized by diarrheagenic *Escherichia coli* which constitute a probable hazard to human health

**DOI:** 10.1371/journal.pone.0266059

**Published:** 2022-04-05

**Authors:** Abeni Beshiru, Anthony I. Okoh, Etinosa O. Igbinosa

**Affiliations:** 1 Applied Microbial Processes & Environmental Health Research Group, Faculty of Life Sciences, University of Benin, Benin City, Nigeria; 2 Stellenbosch Institute for Advanced Study (STIAS), Wallenberg Research Centre at Stellenbosch University, Stellenbosch, South Africa; 3 Department of Environmental Health Sciences, College of Health Sciences, University of Sharjah, Sharjah, United Arab Emirates; 4 SAMRC Microbial Water Quality Monitoring Centre, University of Fort Hare, Alice, Eastern Cape Province, South Africa; Suez Canal University, EGYPT

## Abstract

The study aimed to recover diarrheagenic *Escherichia coli* strains from processed ready-to-eat (RTE) foods in Yenagoa, Nigeria and characterize them using culture-based and molecular methods. Three hundred RTE food samples were collected randomly from different food outlets between February 2021 and August 2021 and assessed for the occurrence of *E*. *coli* using standard bacteriological procedures. The virulence factor formation and antibiotic susceptibility profile of the isolates was carried out using standard microbiological procedures. Polymerase chain reaction (PCR) was used to confirm the identity of the isolates via specific primers and further used to assay the diarrheagenic determinants of the *E*. *coli* isolates. The prevalence of *E*. *coli* positive samples based on the proliferation of *E*. *coli* on Chromocult coliform agar forming purple to violet colonies was 80(26.7%). The population density of *E*. *coli* from the RTE foods ranged from 0–4.3 × 10^4^ ± 1.47 CFU/g. The recovered *E*. *coli* isolates (*n* = 62) were resistant to antibiotics in different proportions such as ampicillin 62(100%), aztreonam 47(75.81%) and chloramphenicol 43(69.35%). All the recovered *E*. *coli* isolates were resistant to ≥ 2 antibiotics. The multiple antibiotic-resistant index (MARI) ranged from 0.13–0.94 with 47(75.8%) of isolates having MARI >2. A total of 48(77.4%) of the isolates were multidrug-resistant (MDR). The proportion of extracellular virulence factor formation is as follows: protease 12(19.35%), curli 39(62.9%), cellulose 21(33.89%), ornithine decarboxylase 19(30.65%) and aesculin hydrolysis 14(22.58%). The overall proportion of diarrheagenic *E*. *coli* was 33/62(53.2%). The distributions of typical diarrheagenic *E*. *coli* includes: tETEC 9(14.5%), tEPEC 13(20.9%), tEAEC 6(9.7%), tEIEC 2(3.2%) and tEHEC 3(4.8%). The proportions of atypical strains include aETEC 10(16.1%), aEAEC 5(8.1%), aEPEC 1(1.6%) and aEIEC 3(4.8%). This study demonstrated that some RTE foods sold in Yenagoa, Nigeria, are contaminated and constitute a probable human health hazard. Thus, there is a need for intensive surveillance of this isolate in RTE foods variety to spot evolving AMR phenotypes and avert food-borne infections.

## Introduction

The consequences and occurrence of food-borne diseases (FBD) have been under-reported, and linking death or illness to food contamination is difficult [1]. *Escherichia coli* (*E*. *coli*) is a typical inhabitant of the gut of warm-blooded animals and is used frequently as an indicator bacterium of faecal contamination in the food industry [2]. *E*. *coli* is a non-spore-forming, Gram-negative rod, usually motile by peritrichous flagella that is a member of the family Enterobacteriaceae [3]. Although most strains are not likely to cause disease, certain *E*. *coli* pathotypes have acquired genetic virulence factors that enable them to generate widespread diseases with increased potential to adjust to new environments [4]. *E*. *coli* expressing virulence traits are capable of causing a variety of disease syndromes via multiple mechanisms and display vast genetic differences within and between pathotypes. *E*. *coli* are designated into pathogenic variants (pathovars, also known as pathotypes) based largely on molecular markers associated with specific disease presentations. Many of these biomarkers are present on mobile genetic elements, which provide difficulties in the accurate identification and surveillance of these pathogens [5]. *E*. *coli* has been associated with human infections including diarrhoea, urinary tract infections, and meningitis. It may also cause acute enteritis in humans as well as animals and is a general cause of ‘traveller’s diarrhoea’ a dysentery-like disease affecting humans, and haemorrhagic colitis often referred to as ‘bloody diarrhoea’ [3].

Diarrheagenic *E*. *coli* (DEC) represent the foremost subgroup tangled in gastrointestinal infection triggered by the consumption of contaminated RTE foods. The strains of DEC have been classified into six diverse pathotypes: enteropathogenic (EPEC), enterotoxigenic (ETEC), Shiga toxin-producing (STEC), enteroinvasive (EIEC), diffusely adherent (DAEC) and enteroaggregative (EAEC). Each pathotype of *E*. *coli* has specific elements answerable for virulence factors coding that host physiology [4]. Among the crucial determinants, *stx* (Shiga toxin gene) has been associated with strains of STEC. STEC represent one of the utmost significant strains, primarily because of their capability to initiate an array of infections. The determinants *lt* (enterotoxin heat-labile) and *st* (enterotoxin heat-stable) are allied with ETEC: the causative agent of diarrhoea in children [6]. EPEC strains encompass the *bfp*A (bundle-forming pilus) and *eae* (intimin) determinants [6]. Food may well aid as the central route for spreading disease-causing *E*. *coli*. Although *E*. *coli* is incapacitated by heat treatments in processed/cooked food, cross-contamination which results from post-processing from the environment and equipment may arise sequel to the pathogen’s perseverance.

The emergence of *E*. *coli* isolates with multiple antibiotic-resistant phenotypes, involving co-resistance to ≥4 unrelated families of antibiotics, has been previously reported and is considered a serious health concern [3]. Transference of resistance determinants by mobile genetic elements including plasmids, transposons, and gene cassettes in integrons and the alteration in locus regulation are important factors that can contribute to the increase in multi-resistant bacteria [7, 8]. Multidrug resistance (MDR) has been increased all over the world that is considered a public health threat. Several recent investigations reported the emergence of MDR bacterial pathogens from different origins including humans, birds, cattle, and fish that increase the need for routine application of the antimicrobial susceptibility testing to detect the antibiotic of choice as well as the screening of the emerging MDR strains [9–16]. Increasing antibiotic resistance among *E*. *coli* contributes to morbidity, mortality, and substantial healthcare and societal costs associated with infection. The high occurrence of antimicrobial-resistant (AMR) strains is linked to the increasingly high and indiscriminate antibiotic usage [17]. Applying these antimicrobials in veterinary medicine at low concentrations for long periods as animal feed additives can culminate in the selection and dissemination of AMR to other bacteria within the food chain. *E*. *coli* has been adopted as a sentinel bacterium for AMR surveillance. *E*. *coli* contaminated food could be an imperative pool for resistance genes or AMR microorganisms. Some research findings in literatures have shown the occurrence of AMR strains of *E*. *coli* in foods [18–20].

Food is one of the most essential and indispensable basic needs of humans required for the nourishment and sustainability of life. The quality of food available and ready for consumption directly impacts the quality of life obtainable in any society. Improved health and higher productivity are achievable without disease, including food-borne illnesses (FBI). Safe food is essential and fundamental for a healthy, productive and reproductive life. Lack of access to safe food causes a destructive cycle of disease, specifically affecting people with ill-health, children, and the elderly [1]. According to the World Health Organization (WHO) report, 550 million people become ill, and 230,000 die yearly due to diarrheal diseases associated with the ingestion of foods already contaminated by microbial pathogens [1]. In another report by World Bank [21], the overall productivity loss linked to FBD in developing countries is estimated to cost $95.2 billion annually, and the amount spent in treating FBI per year is estimated at $15 billion. According to the report, the highest occurrence of food-borne diseases is in Asia and sub-Saharan Africa compared to other continents. Since man’s existence, FBD has been a critical challenge for all nations and people of the world [22].

Food-borne pathogens (FBP) of public health importance such as *E*. *coli* O157:H7, *Salmonella*, *Vibrio*, *Shigella*, *Clostridium* spp. and *Staphylococcus aureus* have been isolated from fresh-cut ready to eat fruits, vegetables and RTE foods sold on the streets, markets, bukaterian, cafeterias, schools, major cities and fast-food restaurants in Nigeria [7, 23–25]. Nigeria, being the most populated country in sub-Saharan Africa, is currently faced with FBD problems usually not reported except in cases of outbreaks. In January 2018, the Nigerian Centre for Disease Control (NCDC) was alerted of a botulism outbreak linked to food consumption in Abuja [26]. Food handlers and peddlers have been connected as sources of pathogens’ contamination of foods. Due to poverty endemic in this region, scarcity and ignorance, many people ingest any available food that satisfies their hunger or quenches their thirst. Safer food is paramount for general well-being and socio-economic growth.

Several factors predispose RTE foods to contamination by microorganisms: low-quality raw materials, lack of proper hygiene and storage in unfitting conditions are the key features triggering heightening the microbial growth and spoilage of hazardous FBP in foods [27]. Food vendors in Nigeria are frequently ill-educated and inexpert in food hygiene. The majority of them prepare foods in unpolished, unhygienic environments with little or no acquaintance about the cause of FBD. In addition, street foods sold in food stands with simple structures and running water are usually not available. Furthermore, suitable washing conveniences and toilets are not the regularly general public. The washing is generally carried out in large bowls or buckets, with rodents and insects on-site without organized sewage disposal. Food is not satisfactorily prevented from flies with refrigeration unavailable. Other factors include unfitting holding temperatures, the old-style processing approaches of food preparation, and individual hygiene of food handlers. Customers who rely on such food are particularly concerned about its ease and usually pay little or no consideration to its quality, hygiene and safety.

There is poor knowledge of food-borne diseases and their transmission among food handlers selling foods in retail outlets and no enforcement from the government in Nigeria. Also, most food vendors in Nigerian are not duly licensed, and their staffs are not correctly selected. Daily, many people are exposed to various kinds of FBI by ingesting the hawked foods. Some symptoms of the food-borne illness include stomach pain, diarrhoea, vomiting, nausea, and headache. Though there has been some conventional microbiological evaluation of *E*. *coli* from RTE processed foods in Nigeria, there is no information on the characterization of diarrheagenic *E*. *coli* from RTE foods in Nigeria. Hence, the need to examine RTE foods for diarrheagenic *E*. *coli* determinants in Yenagoa Nigeria, using conventional and molecular approach to create awareness on the risk of possible FBI among consumers.

## Materials and methods

### Sample collection

The study is focused on RTE foods from Yenagoa, Nigeria. The sample size was determined as follows:

$$\text{Sample}\;\left(\text{N}\right)=\frac{\left({\text{Z}}_{1-\propto /2}\right)²\;\text{P}\left(1-\text{P}\right)}{\;\text{d}²}$$

P = Predictable incidence (5.20–74%) founded on previous studies [27, 28]; Z_1-α/2_ = Typical standard variant at 5% type I error (P < 0.05); d = Complete precision or error (which is 5%). A total of 300 RTE processed food samples were randomly purchased from food outlets in Yenagoa, Nigeria, from February—August 2021 using sterile universal containers. The sample distributions were from restaurants (50 samples), cafeterias (50 samples), street foods (200 samples). The sample discrimination for street food was because it is the most visited and patronized coupled with their ubiquity. All the samples were taped up, labelled and carried in an icebox to the research laboratory for examination within six hours of collection.

### Isolation and identification of *E*. *coli*

The enumeration and isolation of *E*. *coli* isolate from processed RTE food samples was in line with previous procedures [29, 30]. Twenty-five grams (25g) of RTE food samples were homogenized into a sterilized 225 mL of buffered dilution water (Lab M, Lancashire, United Kingdom), culminating in a first-order dilution (10^−1^). The samples were made uniform at 800 rpm for 1 min using a shaker and diluted serially (10^−1^–10^−5^). Each dilution was plated by aseptically transferring 100 μL diluent to 3 plates of Chromocult coliform agar (Merck, Darmstadt Germany). The inoculum was spread over the surface of the agar plate using a sterilized bent glass streaking rod. Plates were retained upright until agar absorbed the inoculum (about 10 min on suitably dried plates). The plates were upturned and incubated for 24 h at 37°C. Purple to violet-coloured colonies of presumptive *E*. *coli* were enumerated and expressed in CFU/g. Isolates were purified on MacConkey agar (Lab M, Lancashire, United Kingdom) and kept in nutrient agar (Lab M, Lancashire, United Kingdom) slants at 4°C until ready for further use.

### Molecular identification of the *E*. *coli* isolates

The biochemical, physiological and morphological characterization of the *E*. *coli* isolates were carried out following the procedures of Lupindu [31] *E*. *coli* isolates that were Gram-negative rod-shaped, catalase-positive, methyl red positive, citrate negative, coagulase-negative, indole positive, oxidase negative, urease negative, Voges Proskauer negative and were able to anaerobically utilize rhamnose, trehalose, xylose, lactose, maltose, mannitol or mannose were screed for the presence of *E*. *coli* via specific primers and polymerase chain reaction (PCR).

### Genomic DNA extraction

The genomic DNA extraction was carried out according to the method of Chen and Kuo [32] with adjustments. Briefly, 3.0mL of an overnight culture was grown in Luria-Bertani (LB) broth at 37°C for 16 h and centrifuged at 27787 × *g* for 3 min. The pellet was transferred into 200μL of lysis buffer (40 mM, pH 7.8, 20 mM sodium acetate, Tris-acetate, 1 mM EDTA, 1% SDS), mixed gently and incubated for 30 min at 37°C. A 50 μL of 5M NaCl suspension was centrifuged at 27787 × *g* for 10 min, followed by mixing the supernatant with 200μL of chloroform and centrifuging for 10 min at 27787 ×*g*. DNA from the upper aqueous phase was precipitated with 200 μL isopropanol, washed with 70% ethanol, dried and finally re-suspended in 50 μL TE buffer (Tris/EDTA buffer with RNase) for PCR. The DNA was stored at -20°C until used.

### Identification and characterization of *E*. *coli* isolates using polymerase chain reaction procedure

For the amplification of the *uidA* gene, the PCR primer pair in the S1 Table in S1 File for the *E*. *coli* specific gene was used to identify the bacteria [33]. The 50 μL PCR approaches which include: 10 μL of gDNA, 5 μL PCR buffer with MgCl_2_, 2.5μL F primer (adjusted to 10 pmol/μL), 2.5 μL R primer (adjusted to 10 pmol/μL), 6 μL dNTP mix, 0.3 μL Taq polymerase and 23.7 μL nuclease-free water, were introduced into the appropriate PCR-tubes. All PCR reactions were pipetted up and down to ensure that everything was well mixed after brief centrifugation. The PCR procedures were carried out using the Peltier-Based Thermal Cycler (Zhengzhou Mingyi Instrument, China) following procedures described previously [33]. For gel electrophoresis, a 1.0% agarose gel was prepared composed of 4 g agarose and 1× 400 mL TAE buffer. For optimal homogenization, the mixture was heated in the microwave and in between shaken several times until all powder residues had dissolved. The gel was then stored at 60°C in the incubator. The gel was prepared in the gel electrophorese chamber. In this case, 1μL GelRed was placed in the agarose gel before the gel polymerized per 100 mL gel. The wells were filled with a mixture of 5 μL PCR products and 2 μL DNA gel loading dye. The gel was run for one hour at a DC voltage of 100V.

### Phenotypic detection of virulence factors

Colonies grown on tryptone soy agar (TSA) (Lab M, Lancashire, UK) were suspended in 3 ml of Mueller-Hinton broth. The density of this suspension was adjusted to 0.5 McFarland standards, the equivalence of 10^6^ cells/mL. Five (5) or 10 mL of this suspension was inoculated on respective plates where applicable for assay of the virulence factor formations and incubated for 24 to 48 h at 37°C. The extracellular protease was assayed following the procedures of [34] on TSA plates supplemented with 1% casein (v/v). The zone of clearance due to casein breakdown was well considered a positive result. The production of curli/fimbriae was determined following the procedures of [35] with modifications using TSA supplemented with 20mg of brilliant blue per litre and 40mg of Congo red per litre. Isolates were grouped into three different morphotypes: rough, dry, and red, (rdar) indicating cellulose and curli/fimbriae production; brown, rough, and dry (bdar) showing a lack of cellulose synthesis but curli/fimbriae production; white and smooth (saw), indicating a lack of both cellulose and curli/fimbriae production [35]. For ornithine, lysine, and arginine assay, a drop of 18–24 h broth culture was inoculated into each of the three decarboxylase broths (ornithine, lysine, and arginine). A 4 mm layer of sterilized mineral oil was included in each of the tubes and incubated at 35–37°C for four days in ambient air and observed for change of colour at 24, 48, 72, and 96 h. A positive test was a purple turbid to yellow-purple faded-out colouration (alkaline) [36].

The lipase assay was assayed on TSA supplemented with 1% Tween 80 (v/v). A clear halo surrounding the areas where the lipase-producing bacterium has proliferated considered a positive result [37]. The bile aesculin agar (Merck, Darmstadt Germany) was inoculated and incubated for 24 h at 37°C. The occurrence of a black halo or dark brown pigmentation/colonies designates a positive test for aesculin hydrolysis [38]. For the beta-galactosidase test (ONPG), the *E*. *coli* were grown in a medium with lactose (to induce the production of the galactosidase enzyme). An aliquot of 0.5 mL of the saline was pipetted into a sterile tube and was inoculated with the bacterium. The ONPG disc was added sterilely (forceps dipped in alcohol and flamed) to the tube and incubated at 37°C for 4 h. The fluid and disc turned yellow for a positive result. The disk (cefinase disk) was dispensed from the cartridge onto a microscope slide using a single disk dispenser for the beta-lactamase test. The disc was moistened with one drop of sterile distilled water. This was followed by removing several well-isolated similar colonies, smeared onto a disk surface, smeared onto a disk surface, and observed for colour change. Yellow to pink-red colour change on the area where the culture was applied indicates a positive result [39].

### Antimicrobial susceptibility profile

The antimicrobial susceptibility profile of the *E*. *coli* isolates was carried out using the Kirby-Bauer disc diffusion method and readings were interpreted by adopting the breakpoints of Clinical and Laboratory Standard Institute. Briefly, purified isolates were inoculated on 5 mL TSB (Lab M, Lancashire, United Kingdom) and incubated overnight. The optical density (OD) of the turbidity of the broth was determined to conform with the OD of the McFarland standard, where the cells are equivalent to 10^6^ cfu/mL. Using sterile swab stick, respective broth cultures were aseptically swabbed on Mueller Hinton agar (Lab M, Lancashire, United Kingdom). A total of 16 antibiotic discs (Mast Diagnostics, Merseyside United Kingdom) which includes nitrofurans [nitrofurantoin (300 μg)], monobactam [aztreonam (30 μg)], macrolides [azithromycin (15 μg)], tetracyclines [tetracycline (30 μg)], folate pathway inhibitor [trimethoprim-sulfamethoxazole (1.25/23.75 μg)], phenicols [chloramphenicol (30 μg)], quinolones [ciprofloxacin (5 μg), nalidixic acid (30 μg)], cephalosporins [ceftazidime (30 μg), cefotaxime (30 μg)], carbapenems [imipenem (10 μg)], aminoglycosides [streptomycin (10 μg), gentamicin (10 μg)], penicillins [ampicillin (10 μg)], β-lactam combination agents [ampicillin/sulbactam (10/10 μg), amoxicillin-clavulanate (20/10 μg)] were employed. The antibiotics were selected based on their clinical relevance to infections caused by *E*. *coli*. The individual discs were impregnated aseptically on the Mueller Hinton agar (Lab M, Lancashire, United Kingdom) plates using sterile forceps equidistant apart. An average of 5 antibiotic discs was impregnated per plate and incubated for 18–24 h at 37°C. Characterization of the isolates’ resistance, intermediate or sensitivity profile was evaluated by measuring inhibition zones and compared with the interpretative chart using the Clinical and Laboratory Standard Institute [40]. Multiple antibiotic resistance index, extensively drug-resistant (XDR), pan drug-resistant (PDR) and multidrug resistance (MDR) was evaluated following previous procedures [41, 42].


$$\text{Multiple}\;\text{antibiotic}\;\text{resistance}\;\text{index}=\frac{\text{Number}\;\text{of}\;\text{the}\;\text{antibiotics}\;\text{to}\;\text{which}\;\text{resistance}\;\text{occurred}}{\text{Total}\;\text{number}\;\text{of}\;\text{antibiotics}\;\text{to}\;\text{which}\;\text{the}\;\text{isolates}\;\text{were}\;\text{tested}}$$

### PCR detection of diarrheagenic determinants

PCR based detection of diarrheagenic virulence determinants was done for all 62 isolates of *E*. *coli*. Virulence determinants such as *aai*, *aat* (EAEC); *eae*, *bfp* (EPEC); *lt*, *st* (ETEC) linked with DEC pathotypes were used to distinguish the pathotypes using PCR amplification procedures. Specific primers involving aggR-activated island (*aaiC*), heat-labile (*lt*), anti-aggregation protein transporter (*aat*), attaching and effacing (*eae*), bundle forming pilus (*bfp*) and heat-stable (*st*) were used to distinguish the individual elements using PCR setup [43–46]. The PCR was done following cycling conditions with an annealing temperature of 57°C for 20 s. As previously described, a different PCR was carried out for Shiga toxin genes (*stx2* and *stx1*) [47, 48]. PCR for invasion associated locus (*ial*) and invasion plasmid antigen H (*ipaH*) was carried out following protocols described previously [49–51]. Primers used are presented in S1 Table in S1 File.

### Data analysis

The data were checked for completeness and accuracy. It was coded, and analysis was done using SPSS version 20 statistical software programme (IBM Corp, Armonk, NY, USA). Descriptive statistics of bacteriological prevalence was presented in mean, standard deviations, frequency tables and percentages with their corresponding 95% Confidence Intervals (Cis). The prevalence results were presented using appropriate cross-tabulations. One Way Analysis of Variance was also used to compare multiple variables. Statistical significance was considered at the *p*-value significant level set at < 0.05.

## Results

### Prevalence and *E*. *coli* counts from RTE foods

The prevalence of *E*. *coli* positive samples from processed RTE foods was 80/300(26.7%). The distribution of the prevalence was restaurant 2/50(4.0%), cafeteria 5/50(10%), street foods 73/200(36.5%). The least prevalence, which is 0, was obtained from Ogbono soup, while the highest prevalence, which is 11(61.1%), was recorded from the vegetable soup. The population density *E*. *coli* of the RTE foods ranged from 0–4.3 × 10^4^ ± 1.47 CFU/g. Other than Ogbono soup, some different food types were contaminated with *E*. *coli* (Table 1). A significant proportion of the *E*. *coli* contaminated foods were recorded from the street food samples. All *E*. *coli* isolates were Gram-negative rod-shaped, catalase-positive, methyl red positive, citrate negative, coagulase-negative, indole positive, oxidase negative, urease negative, Voges-Proskauer negative and were able to anaerobically utilize rhamnose, trehalose, xylose, lactose, maltose, mannitol or mannose. Sixty-two (62) isolates of *E*. *coli* were confirmed using PCR via specific primers and were further characterized for antibiotic susceptibility and virulence attributes.

**Table 1:** Prevalence and *E*. *coli* counts from RTE foods.

Type of sample collected	Number of samples examined	Prevalence of *E*. *coli* positive samples	The population cell density of *E*. *coli*
			CFU/g
Oil and garri soup	17	7(41.2)	4.6 × 10^2^ ± 1.04^b^
Cocoyam soup	18	9(50)	2.1 × 10^3^ ± 1.20^c^
Pepper soup	17	1(5.9)	1.6 × 10^1^ ± 0.10^a^
Plantain porridge	15	2(13.3)	5.4 × 10^2^ ± 1.61^b^
Yam porridge	19	3(15.8)	4.8 × 10^2^ ± 1.06^b^
Fried beans	15	2(13.3)	3.1 × 10^2^ ± 1.23^b^
Porage beans	18	4(22.2)	2.5 × 10^2^ ± 1.42^b^
Jollof rice	18	6(33.3)	6.1 × 10^3^ ± 1.36^c^
Fried rice	15	2(13.3)	2.2 × 10^1^ ± 0.04^a^
Banga rice	15	1(6.7)	1.7 × 10^1^ ± 0.02^a^
Coconut rice	15	3(20)	3.8 × 10^2^ ± 0.07^b^
Bitter leave soup	17	2(11.8)	3.4 × 10^1^ ± 1.09^a^
Okazi soup	18	8(44.4)	2.2 × 10^3^ ± 1.19^c^
Vegetable soup	18	11(61.1)	4.3 × 10^4^ ± 1.47^d^
Melon soup	18	10(55.6)	2.8 × 10^3^ ± 1.24^c^
Okro soup	15	6(40)	3.6 × 10^3^ ± 0.19^c^
Ogbono soup	15	0	-
Banga soup	17	3(17.6)	3.4 × 10^1^ ± 0.18^a^
Total	300	80(26.7)	

**Legend**: Mean population cell density is expressed in mean ± standard deviation of the mean. Mean with significant difference carry different alphabets

### Antibiotic susceptibility profile of the *E*. *coli* isolates

The *E*. *coli* isolates in Table 2 were resistant to antibiotics in different proportions such as ampicillin 62(100%), aztreonam 47(75.81%), chloramphenicol 43(69.35%), ciprofloxacin 38(61.29%), tetracycline 39(62.90%), ampicillin/sulbactam 37(59.68%) and azithromycin 36(58.06%). They were also sensitive to imipenem 61(98.39%), nitrofurantoin 52(83.87%), gentamicin 33(53.23%), streptomycin 32(51.61%) and azithromycin 26(41.94%).

**Table 2:** Antimicrobial susceptibility profile of the *E*. *coli* isolates.

Antimicrobial class	Antibiotics	*E*. *coli* (*n* = 62)		
		Sensitive	Intermediate	Resistance
β-Lactam combination agents	Ampicillin/sulbactam (10/10 μg)	6(9.68)	19(30.65)	37(59.68)
	Amoxicillin-clavulanate (20/10 μg)	9(14.51)	24(38.71)	29(46.77)
Penicillins	Ampicillin (10 μg)	0	0	62(100)
Aminoglycosides	Gentamicin (10 μg)	33(53.23)	20(32.26)	9(14.51)
	Streptomycin (10 μg)	32(51.61)	14(22.58)	16(25.81)
Carbapenems	Imipenem (10 μg)	61(98.39)	1(1.61)	0
Cephalosporins	Cefotaxime (30 μg)	9(14.52)	19(30.65)	34(54.84)
	Ceftazidime (30 μg)	8(12.90)	22(35.48)	31(50)
Quinolones	Nalidixic acid (30 μg)	5(8.07)	28(45.16)	29(46.77)
	Ciprofloxacin (5 μg)	5(8.07)	19(30.65)	38(61.29)
Phenicols	Chloramphenicol (30 μg)	6(9.68)	13(20.97)	43(69.35)
Folate pathway inhibitor	Trimethoprim-sulfamethoxazole (1.25/23.75 μg)	24(38.71)	17(27.42)	21(33.87)
Tetracyclines	Tetracycline (30 μg)	10(16.13)	13(20.97)	39(62.90)
Macrolides	Azithromycin (15 μg)	26(41.94)	NA	36(58.06)
Monobactam	Aztreonam (30 μg)	6(9.68)	21(33.87)	47(75.81)
Nitrofurans	Nitrofurantoin (300 μg)	52(83.87)	7(11.29)	3(4.84)

### Multiple antibiotic resistance index (MARI) and multidrug resistance (MDR) profile of the *E*. *coli* isolates

The most predominant resistance phenotype was AZM^R^, AZT^R^, TET^R^, CHL^R^, CIP^R^, NAL^R^, CAZ^R^, CTX^R^, AMP^R^, AMS^R^, AMC^R^ for 20(32.3%) and AZM^R^, AZT^R^, TET^R^, CHL^R^, CIP^R^, NAL^R^, CAZ^R^, CTX^R^, AMP^R^, AMS^R^, AMC^R^, STX^R^, STR^R^ for 10(16.1%) of the isolates of *E*. *coli* (Table 3 and S2a Table in S1 File). Furthermore, a single isolate (PES080) from pepper soup (street food) was MDR to 15 antibiotics (AZM^R^, AZT^R^, TET^R^, CHL^R^, CIP^R^, NAL^R^, CAZ^R^, CTX^R^, AMP^R^, AMS^R^, AMC^R^, STX^R^, NIT^R^, STR^R^, GEN^R^) with MARI of 0.94 (Table 3 and S2a Table in S1 File). All the recovered *E*. *coli* strains were resistant to ≥2 antibiotics. The MARI of the isolates ranged from 0.13–0.94, with 47(75.8%) of the isolates having MARI >2. A total of 48(77.4%) of the isolates were MDR (S2a Table in S1 File). The distribution of the resistance to antibiotics and their MARI with respect to their sample source includes restaurant [resistance to 2–3 antibiotics (MARI: 0.13–0.19)], cafeteria [resistance to 2–6 antibiotics (MARI: 0.13–0.38)], street food [resistance to 2–15 antibiotics (MARI: 0.13–0.94)]. A total of 11/62(17.7%) isolates were extensively drug-resistant (non-susceptible to ≥1 agent in all but ≤ 2 categories) while none of the isolates was pan drug-resistant (non-susceptible to all antimicrobial agents used). The extensively drug-resistant isolates had MARI, ranging from 0.81–0.94 (S2a Table in S1 File).

**Table 3:** Multidrug resistance (MDR) and multiple antibiotic resistance index (MARI) profile of the *E*. *coli* isolates.

No of antimicrobial class	No of antibiotics	Resistance phenotypes	No of *E*. *coli* isolates (*n* = 62)	MARI
11	15	AZM^R^, AZT^R^, TET^R^, CHL^R^, CIP^R^, NAL^R^, CAZ^R^, CTX^R^, AMP^R^, AMS^R^, AMC^R^, STX^R^, NIT^R^, STR^R^, GEN^R^	1(1.6)	0.94
10	13	AZM^R^, AZT^R^, TET^R^, CHL^R^, CIP^R^, NAL^R^, CAZ^R^, CTX^R^, AMP^R^, AMS^R^, AMC^R^, STX^R^, STR^R^	10(16.1)	0.81
9	12	AZM^R^, AZT^R^, TET^R^, CHL^R^, CIP^R^, NAL^R^, CAZ^R^, CTX^R^, AMP^R^, AMS^R^, AMC^R^, NIT^R^	2(3.2)	0.75
8	11	AZM^R^, AZT^R^, TET^R^, CHL^R^, CIP^R^, NAL^R^, CAZ^R^, CTX^R^, AMP^R^, AMS^R^, AMC^R^	20(32.3)	0.69
8	9	AZM^R^, AZT^R^, TET^R^, CHL^R^, CIP^R^, CAZ^R^, CTX^R^, AMP^R^, AMS^R^	8(12.9)	0.56
6	7	AMP^R^, NAL^R^, CIP^R^, CHL^R^, TET^R^, AZT^R^, AZM^R^	2(3.2)	0.44
5	6	AMP^R^, GEN^R^, STR^R^, CHL^R^, AZT^R^, AZM^R^	2(3.2)	0.38
3	4	AMS^R^, AMC^R^, AMP^R^, AZM^R^	3(4.8)	0.25

**Legend:** AMS: Ampicillin/sulbactam (10/10 μg), AMC: Amoxicillin-clavulanate (20/10 μg), AMP: Ampicillin (10 μg), GEN: Gentamicin (10 μg), STR: Streptomycin (10 μg), IMI: Imipenem (10 μg), CTX: Cefotaxime (30 μg), CAZ: Ceftazidime (30 μg), NAL: Nalidixic acid (30 μg), CIP: Ciprofloxacin (5 μg), CHL: Chloramphenicol (30 μg), STX: Trimethoprim-sulfamethoxazole (1.25/23.75 μg), TET: Tetracycline (30 μg), AZT: Azithromycin (15 μg), AZM: Aztreonam (30 μg), NIT: Nitrofurantoin (300 μg)

### Extracellular virulence factor formation of the *E*. *coli* isolates

**Fig 1:**
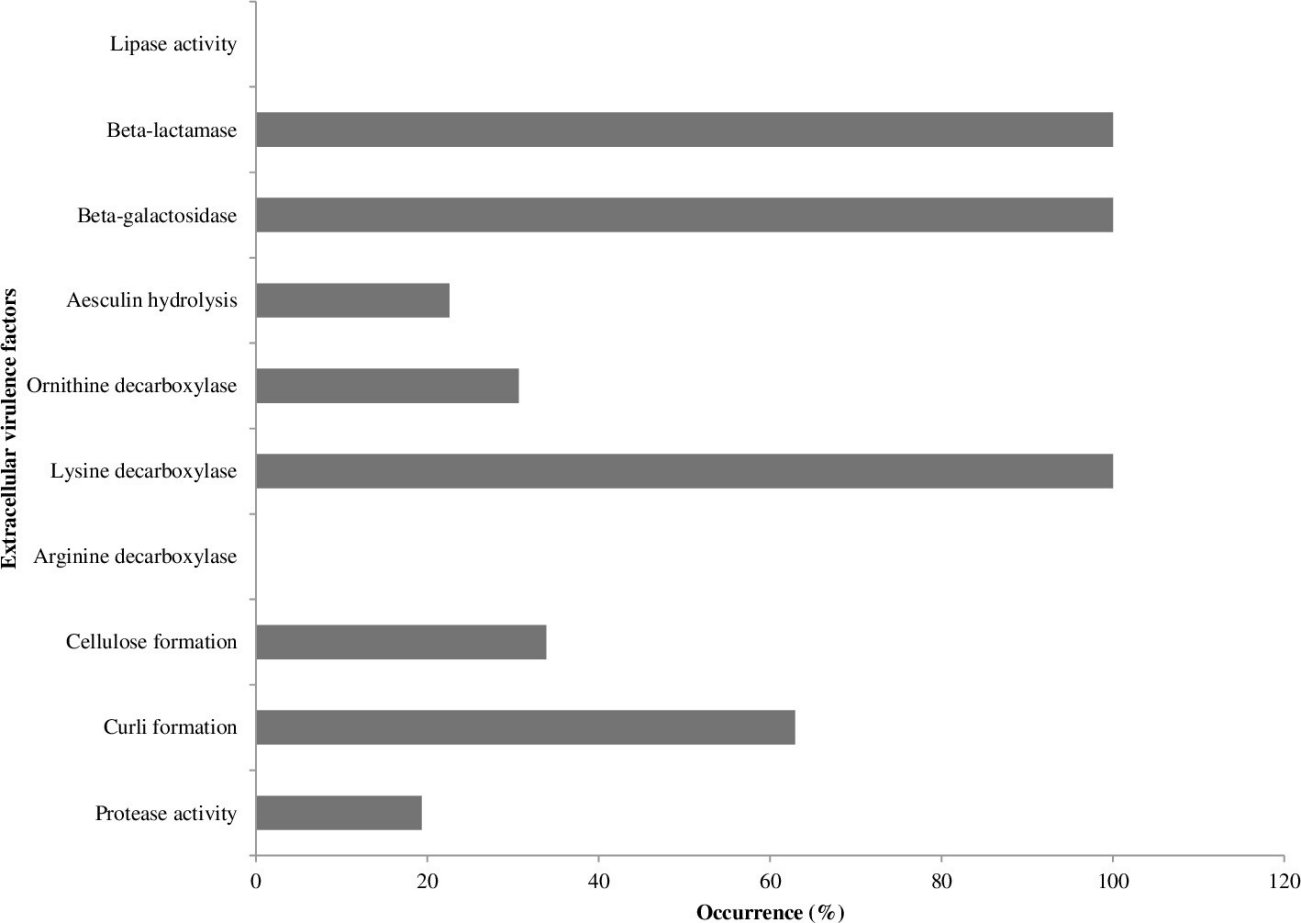
Extracellular virulence factor formation of the *E*. *coli* isolates.

The proportion of extracellular virulence factor formation in Fig 1 is as follows: protease activity 12(19.35%), curli formation 39(62.9%), cellulose formation 21(33.89%), lysine decarboxylase activity 62(100%), ornithine decarboxylase activity 19(30.65%), aesculin hydrolysis activity 14(22.58%), beta-galactosidase activity 62(100%) and beta-lactamase activity 62(100%). The isolates were all negative for lipase and arginine decarboxylase activity (S2b Table in S1 File). Protease activity significantly correlated cellulose formation (*r* = 0.426, *p*<0.01) and ornithine decarboxylase (*r* = 0.737, *p*<0.01). Curli formation significantly correlated cellulose formation (*r* = 0.550, *p*<0.01) and aesculin hydrolysis (*r* = 0.335, *p*<0.01). Cellulose formation significantly correlated ornithine decarboxylase (*r* = 0.263, *p*<0.05) and aesculin hydrolysis (*r* = 0.347, *p*<0.01) (S3a Table in S1 File).

### Diarrheagenic profile of *E*. *coli* from processed RTE foods

**Fig 2:**
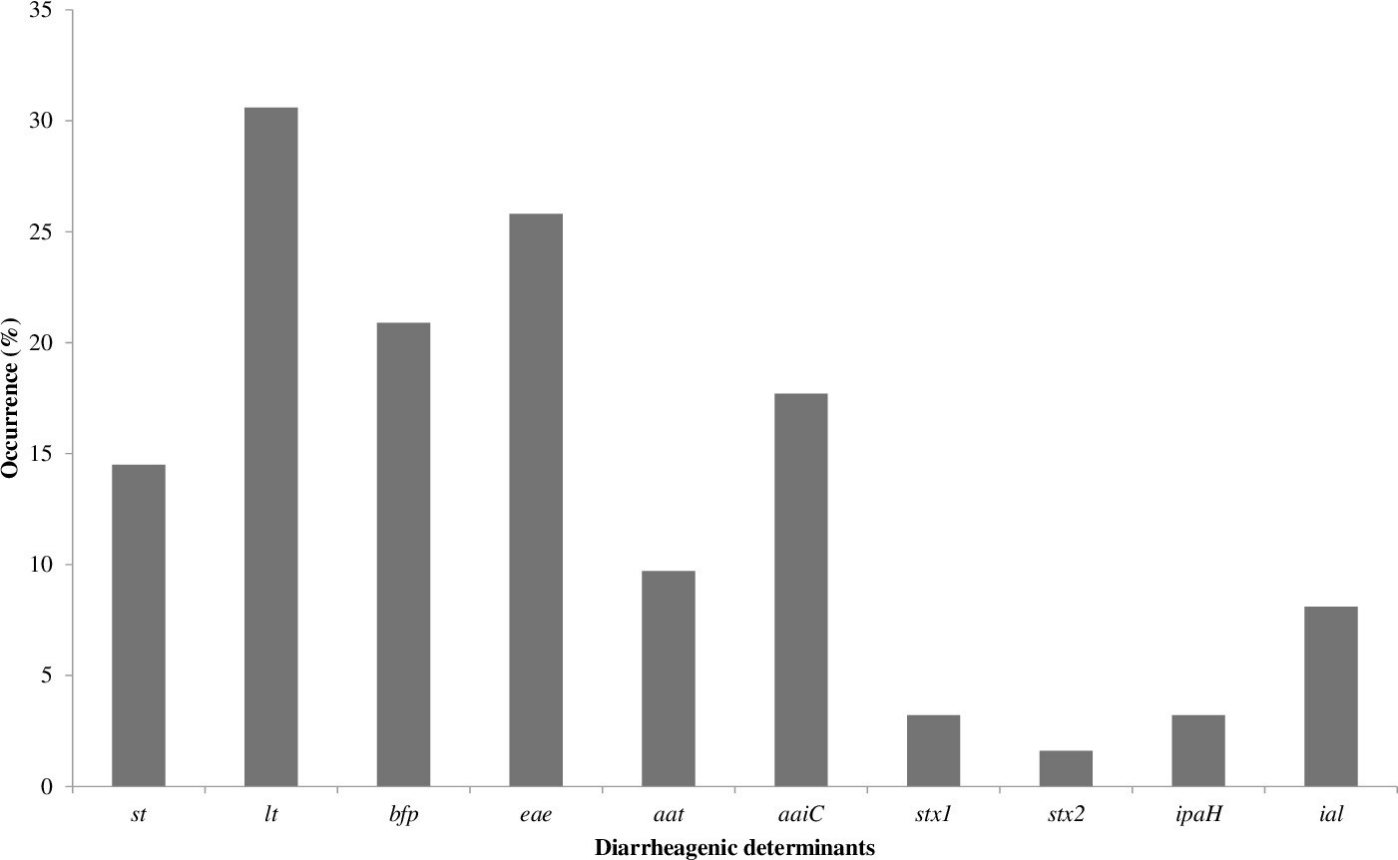
Diarrheagenic determinants of *E*. *coli* from processed RTE foods.

**Fig 3:**
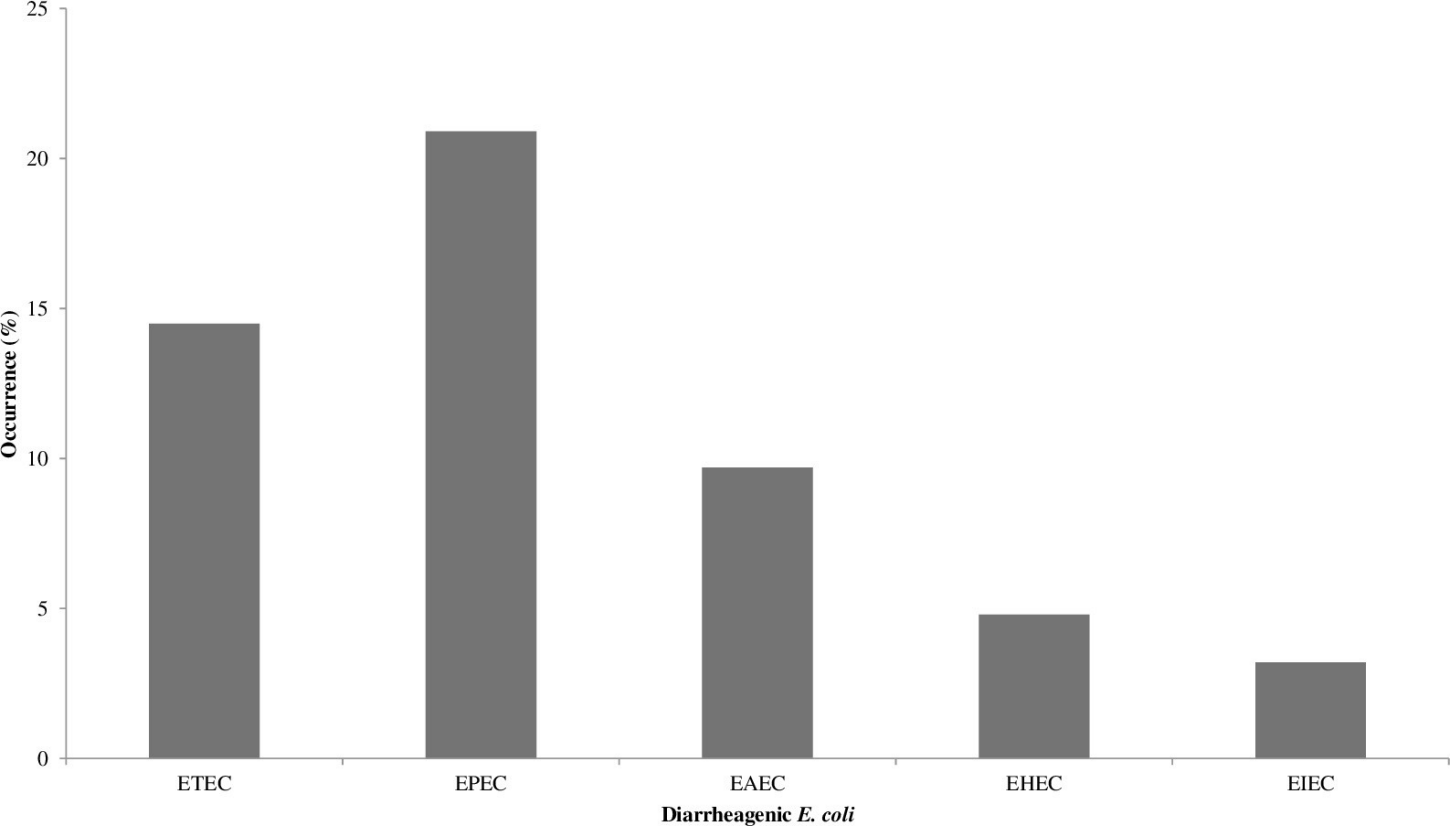
Typical diarrheagenic *E*. *coli* from processed RTE foods.

The proportions of diarrheagenic determinant of *E*. *coli* from processed RTE food is as follows: *st* 9(14.5%), *lt* 19(30.6%), *bfp* 13(20.9%), *eae* 16(25.8%), *aat* 6(9.7%), *aaiC* 11(17.7%), *stx1* 2(3.2%), *stx2* 1(1.6%), *ipaH* 2(3.2%) and *ial* 5(8.1%) (Fig 2 and S3b Table in S1 File). The proportions of typical diarrheagenic *E*. *coli* in Fig 3 includes: tETEC 9(14.5%), tEPEC 13(20.9%), tEAEC 6(9.7%), tEIEC 2(3.2%) and tEHEC 3(4.8%). The proportions of atypical diarrheagenic *E*. *coli* strains include aETEC 10(16.1%), aEAEC 5(8.1%), aEPEC 1(1.6%) and aEIEC 3(4.8%). There were no atypical strains for EHEC. The overall proportion of diarrheagenic *E*. *coli* was 33/62(53.2%). A total of 12 isolates had no diarrheagenic determinant, while 50(80.6%) isolates had ≥1 diarrheagenic determinant (S2b Table in S1 File). ETEC isolates showed increased curli formation, cellulose formation and aesculin hydrolysis. The aEAEC isolates showed poor curli and cellulose formation (S2b Table in S1 File).

Isolates from a particular sample source (street foods) showed increased propensity to be resistant to multiple antibiotics (*r* = 0.663, *p*<0.01), to possess *lt* gene (*r* = 0.377, *p*<0.01), form curli (*r* = 0.586, *p*<0.01), form cellulose (*r* = 0.354, *p*<0.01), and hydrolyse aesculin (*r* = 0.307, *p*<0.05) (S3b Table in S1 File). Isolates that possessed the following showed high tenacity to be resistant to multiple antibiotics: *st* gene (*r* = 0.463, *p*<0.01), *lt* gene (*r* = 0.647, *p*<0.01), protease activity (*r* = 0.336, *p*<0.01), curli formation (*r* = 0.767, *p*<0.01), cellulose formation (*r* = 0.751, *p*<0.01), aesculin hydrolysis (*r* = 0.448, *p*<0.01) (S3b Table in S1 File).

## Discussion

The RTE foods are considered as high-risk foods since no additional action such as re-heating is required before eating. Improper handling of RTE food may cause contaminations, and display at improper temperature favours the rapid growth of pathogens and may result in foodborne outbreaks. Thus, regulation of bacteria concentration is crucial to guarantee the safety of RTE foods. Our study shows that >¼th of RTE foods assessed from Yenagoa, Nigeria had a poor microbiological quality was far lower than the 72.3–85% report from Mexico [2, 52], 69% from Iran [53] and 39.2% from China [54]. Much lower reports compared to the findings from our study have been reported, such as 20.83% in North Africa [55], 8.3% in Vietnam [18], 3.1% in South America [4]. Comparisons of the *E*. *coli* densities of RTE foods from retail food outlets in Yenagoa showed noteworthy differences (*p* < 0.05), signifying that the density of *E*. *coli* in RTE foods from Yenagoa Nigeria was affected by the different cuisine. More *E*. *coli* contaminated RTE processed food samples were recorded from the street food samples compared to the food samples from restaurants and cafeterias. This could be attributed to poor hygiene practized during food handling, cross-contamination, as well as time and temperature control.

The high prevalence of 11(61.1%) of *E*. *coli* contaminated vegetable soup in this study could further be ascribed to its processing (minimally processed) to retain the quality of nutrients and enhance palatability to the consumers. Thus, vegetables harbouring *E*. *coli* could be traced to the farming activities via human and animal waste manure and wastewater/untreated water used for irrigation or various processing stages such as slicing and peeling, favouring its interaction with *E*. *coli*. The inability of *E*. *coli* to recover from Ogbono soup and poor recovery rate from pepper soup, coconut rice, and Banga rice could be ascribed to the cuisine’s oil rancidity and dense nature. The occurrence of *E*. *coli* exceeding the 10^2^ CFU/g limits in some samples calls for concern since *E*. *coli* is well-defined by European Union (EU) bacteriological standards as the pointer bacteria for faecal pollution during processing. Findings highlight the necessity for enhancements in hygiene during production and raw materials selection in processing. Bautista-De-Leon et al. [52] reported that *E*. *coli* density was <3–1100 MPN/g. Luu-Thi and Michiels [18] reported counts which ranged from below detection limit to 6.2 × 10^3^. These detection rates were similar to the findings from our study. Outbreaks associated with RTE foods were documented in Europe, and dissemination of disease-causing microbes due to international food trades couldn’t be eliminated [56].

The difference in the prevalence of *E*. *coli* in human food appears to result from essentially different methods of occurrence valuation. Many studies on prevalence have been reported with strains randomly selected from selective media and preceding investigations [57]. This could have given rise to an underestimation of actual *E*. *coli* prevalence. Here, Chromoccult coliform agar (Merck, Darmstadt Germany) was used, known for its exceptional recovery rates [29, 30]. As the RTE food samples examined were made up of diverse ingredients, antibiotic-resistant *E*. *coli* might not solitarily emanate from RTE foods but also vegetables or spices and condiments that are included to add flavour and aroma to the food. Hence, the precise source of elements inhabiting those RTE foods is challenging to track. Thus, strict sanitation measures during preparation/ handling are of extreme significance to reduce further pathogenic pathotypes of *E*. *coli* spread to the consumers.

Cellulose production may not be intricate in bacterial pathogenesis; however, cellulose-deficient mutants have shown decreased persistence and enhanced sensitivity to AMR treatment regimens in the food environment. *E*. *coli* curli/fimbriae adherences were first carried out on ETEC from porcine calves followed by humans. The determinants coding for the diverse curli/fimbriae can be plasmid-borne, particularly in ETEC or chromosome-borne on other pathotypes of *E*. *coli*. The Bundle-Forming Pili (*bfp*), encoded by plasmid-borne determinants via tEPEC, is alleged to aid bacteria adherence resulting in micro-colonies formation on the host cells. Curli encoded via chromosome-borne elements are formed by various pathogenic strains of *E*. *coli*. Curli may facilitate the fastening of *E*. *coli* to the extracellular matrix of the mucosal post fragilisation of the epithelium; and biofilm formation on biotic and non-biotic surfaces.

Different specific outer membrane lipopolysaccharides and proteins (esculin hydrolysis, β-lactamase, β-galactosidase, lysine-ornithine decarboxylase, protease, cellulose formation) of disease-causing *E*. *coli* strains are involved in membrane disruption, affecting essential phagocytic functions, antimicrobial activity, persistence, bacteria adherence (bacteria-bacteria adherence, bacteria-eukaryotic cell adherence), and biofilm formation. These factors could further contribute to copious adherence to the intestinal mucosa, amplifying cytotoxins and enterotoxins; inducing mucosal swelling; breakdown of host matrix components or meddlesome with host cell-signalling short-circuit processes in host cells. The *eae* gene is situated on a pathogenicity island in conjunction with other determinants coding for effacing and attaching lesions produced by EHEC and EPEC. *E*. *coli* harbours diverse oligopeptide toxins, such as heat-stable enterotoxins. Besides the *st* oligopeptide enterotoxins, human and animal ETEC strains can also yield *lt*.

The usual preparation of RTE foods very rapidly as “by hand” is one source of cross-contamination. This might aid extreme colonization with pathogenic bacteria, increasing the likelihood of conveying AMR and virulence determinants such as diarrheagenic elements. An infection caused by DEC is a global problem and are frequently linked to the ingestion of contaminated water and foods. Processed RTE foods are often directly consumed, which potentially increases the risk of infection-related with the ingestion of these foods. Most food samples in our study generally underwent a thermal treatment regimen at different time intervals, which can hinder most bacteria. The other incidence rates of DEC could be linked to sample number, sample type, quality of sanitation, and geographical location. Hence, DEC in the food can be linked to cross-contamination during handling and processing procedures such as slicing, storing, packaging, and weighing.

In a study by Zhang et al. [54], ETEC harbouring the *st* determinants were the utmost prevalent, compared to *lt*+*st* and *lt*, which were unswerving with reports documented in Japan [58], unlike that of Peru (*lt* 52%, *lt*+*st* 23% and *st* 25%) [59]. The lt was more prevalent in our study, while the *st* and *lt+st* were the same. The overall pathotypes arrangement in products from meat was STEC>EPEC>EAEC>ETEC, and STEC>EAEC>EPEC>ETEC in cheese and raw milk with no strain of EIEC detected [53]. The pathotype arrangement in our study is EPEC>ETEC>EAEC>EIEC/EHEC. ETEC strains that were isolated were lt positive with *st* absent in them; also, all the recovered EPEC strains fitted to atypical strains with *eaeA* gene only [53], which was different from our study. Typical and atypical EAEC strains were also recovered [53], harmonizing with our findings. Identified diarrheagenic *E*. *coli* by Bautista-De-Leon et al. [52] included STEC, ETEC and EPEC from 1.4% of the samples where only STEC strains with *stx1* locus was isolated. This was lower than our study prevalence in diarrheagenic *E*. *coli* where both *stx1* and *stx2* were detected.

Studies showed that tEPEC diarrhoea cases had been substituted with aEPEC in industrialized and developing countries [6]. A study documented the occurrence of tEPEC and aEPEC as 3% and 11%, respectively [60]. The aEAEC is linked with outbreaks of FBI. Alizade et al. [60] showed the ETEC detected as frequent pioneer agents of diarrhoea. ETEC has been documented as the major pathogen accountable for traveller’s diarrhoea and a primary root of mortality and morbidity in children resident in developing countries and travellers going to /and from these destinations. ETEC has also been documented as the leading cause of diarrhoea in <5-year-old kids [60]. The recovery of ETEC in food and food products has been reported in Mexico, Iran, Colombia, and Canada [53].

Concerning the recovery rate of ETEC in our study, it is safe to state that RTE ETEC-contaminated foods are readily available in retail food outlets of Yenagoa, Nigeria and may constitute travellers’ diarrhoea risk to consumers. Castro-Rosas et al. [2] detected EIEC in RTE salads. Mokhtar and Karmi [55] reported that 24% of their *E*. *coli* isolates were EHEC (*stx2*+*eae*) with none of the entire isolates with *stx1* gene, while other 64% of the isolates had the *eae* gene only, which was very high compared to the result of our study; also differ since both Shiga toxin gene was detected in 1 of our isolates. Lima et al. [4] reported that none of the isolates had *eae*, *lt1*, *stx1*, and *stx2* genes detected in ours. In the study by Sivakumar et al. [20], only 11.90% of isolates carried *eae* (aEPEC); 21.42% had *lt* gene (aETEC), with none having *stII* or *bfp* genes; which were a bit different compared to the findings of this present study. Sánchez et al. [19] reported *stx2* (61.1%) being the predominant *stx* subtype detected while *stx1* was our predominant *stx* subtype detected. It is stated that *stx2* and *stx1* produce different types and degrees of damage to the tissue, with *stx2* being more toxic than *stx1* to the renal endothelial cells of humans [52]. ETEC has been recovered from carrot juice and vegetable salads [2]. In developed countries, infections from tEPEC have been reduced, and infections of aEPEC seem to be on the rise recently.

The isolates of Zhang et al. [54] portrayed high resistance to ampicillin, trimethoprim /sulfamethoxazole, and tetracycline, similar to our study. The high resistance in these isolates can be linked with the widespread application of these antibiotics in livestock production and human medicine. More than 50% of the *E*. *coli* strains from this study were cefotaxime-resistant, harmonizing findings from other countries [6, 61]. Our study also showed that ETEC isolates were likely MDR with adherent/persistent characteristics coupled with esculin hydrolysis. In our study, 69.35% of *E*. *coli* isolates were resistant to chloramphenicol, suggesting that unauthorized and irregular usage may have happened. Notably, 77.4% of the *E*. *coli* isolates in our study were MDR, which is bothersome. MDR of 85.71% by Sivakumar et al. [20] was higher than ours. Isolates from Lima et al. [4] showed resistance to 9/15 antibiotics tested, compared to where a particular isolate from our study showed resistance to 15/16 antibiotics tested. However, high resistance found for tetracycline, sulfamethoxazole/trimethoprim, chloramphenicol, and ampicillin by Lima et al. [4] were similar to ours. The increased prevalence of MDR *E*. *coli* epidemic pathotypes in the studied community proposes the necessity for expanding our acquaintance of the reservoirs, transmission pathways and sources.

*E*. *coli* isolates (24.2%) studied by Guo et al. [62] were resistant to ≥1 antimicrobial tested, which was lower compared to ours where all the isolates were resistant to ≥2 antimicrobial tested. Resistance to ampicillin (15.2%), chloramphenicol (10.1%) and tetracycline (17.2%) were the most common [62], which was similar to our findings. All *E*. *coli* isolates by Guo et al. [62] were sensitive to amoxicillin/clavulanic acid, meropenem and amikacin, which were different to our study where resistance was recorded for the antibiotics with the exemption of carbapenem. Although AMR patterns from one country to another differed, resistance to ampicillin, tetracycline, trimethoprim-sulfamethoxazole and streptomycin are the most prevalent compared to other antimicrobials. The MAR index from Fallah et al. [53] was 0.75, compared to our study (0.13–0.94). The existence of specific MDR phenotypes in diverse samples designates cross-contamination amid production lines (disinfection, handling, washing, slicing) or a shared material source (procurement from similar farms or markets). In a previous study, *E*. *coli* were less resistant to ceftazidime than cefotaxime, irrespective of the sample source or type [57], similar to our result. *E*. *coli* resistant to carbapenem have been documented. However, no isolate from our study was carbapenem-resistant. Kim et al. [61] reported all their isolates to be susceptible to amikacin and tigecycline, which was different from our study as proportions of our isolates were aminoglycoside-resistant.

Bacteria resistance to antibiotics is linked essentially to their application as prophylactics or growth promoters [17]. This singularity is a global menace that threatens public health. *E*. *coli* isolates portrayed intermediate resistances to sulfamethoxazole /trimethoprim, gentamicin, cefotaxime, aztreonam and ciprofloxacin. Intermediate resistance implies future resistance, which makes such isolates awkward to handle and must be eliminated by pathogens. Therefore, the high prevalence of *E*. *coli* may indicate a general lack of adherence to hygienic procedures, which are simple but effective measures to reduce the transfer of faecal bacteria. Unfortunately, as we have observed in the restaurants and retail food outlets, hand washing is often neglected by food handlers, particularly with street food handlers, where handwashing is almost wholly neglected. Continuous food safety and hygiene reminders should adhere and to curtail the risk of contamination by FBP in RTE food. This will assist in decreasing the risk of AMR bacterial contamination, which might be crucial as AMR determinant pools and constituting parts of the resistome in the food and food processing environment. Therefore, further investigations are needed for PCR-based detection of antimicrobial resistance genes and ongoing surveillance of the spread of antibiotic resistance in epidemiological and environmental studies. This calls for studies to determine the extent to which transmission of antibiotics-resistant *E*. *coil* occurs and how such transfer impacts the efficacy of anti-*E*. *coil* strains used in human medicine becomes imperative.

## Conclusions

Our study suggests that most pathogenic *E*. *coli* strains isolated from RTE foods possess potential virulence traits. These data should be valuable for assessing human health risks due to the consumption of RTE foods from the community impacted by inadequate cooking or post-processing contamination. This study thus emphasized the need for intensive surveillance of this isolate in RTE foods variety to avert FBI and spot emerging AMR phenotypes since AMR strains may colonize the human population via these RTE foods. Our results also underscore the necessity to sternly control the usage of antimicrobial therapy in human health and agricultural sectors in Nigeria.

## Supporting information

S1 File Supplementary tables. **S1 Table.** PCR primers used in this study for *E*. *coli* detection and virulence genes.; **S2a Table.** Phenotypic profile of *E*. *coli* isolates.; **S2b Table**. Phenotypic and genotypic characterization of *E*. *coli* isolates.; **S3a Table.** Correlation of phenotypic characteristics of *E*. *coli*.; **S3b Table.** Correlation matrix of phenotypic and genotypic characteristics of *E*. *coli*.
(DOCX)
