# Supplementary material for: Processed ready-to-eat (RTE) foods sold in Yenagoa Nigeria were colonized by diarrheagenic Escherichia coli which constitute a probable hazard to human health
Source: PLoS One. 2022 Apr 5;17(4):e0266059. doi: 10.1371/journal.pone.0266059 (PMC8982850; doi:10.1371/journal.pone.0266059)
Supplement: S1 File — S1 Table. PCR primers used in this study for E. coli detection and virulence genes.; S2a Table. Phenotypic profile of E. coli isolates.; S2b Table. Phenotypic and genotypic characterization of E. coli isolates.; S3a Table. Correlation of phenotypic characteristics of E. coli.; S3b Table. Correlation matrix of phenotypic and genotypic characteristics of E. coli. (DOCX) [file pone.0266059.s001.docx]

**Processed ready-to-eat (RTE) foods sold in Yenagoa Nigeria were colonized by diarrheagenic *Escherichia coli* which constitute a probable hazard to human health**

Abeni Beshiru^1,2, ¶^, Anthony I. Okoh^3,4^ and Etinosa O. Igbinosa^1,2,4, ¶^*

^1^Applied Microbial Processes and Environmental Health Research Group, Faculty of Life Sciences, University of Benin, Benin City, Nigeria

^2^ Stellenbosch Institute for Advanced Study (STIAS), Wallenberg Research Centre at Stellenbosch University, Stellenbosch, South Africa

^3^ Department of Environmental Health Sciences, College of Health Sciences, University of Sharjah, Sharjah, United Arab Emirates

^4^ SAMRC Microbial Water Quality Monitoring Centre, University of Fort Hare, Alice, Eastern Cape Province, South Africa

***** Corresponding author E-mail: [etinosa.igbinosa@uniben.edu](mailto:etinosa.igbinosa@uniben.edu%20) (EOI)

^¶^These authors contributed equally to this work

**Running title**: Diarrheagenic multidrug-resistant *E. coli* from locally processed Nigerian foods

**S1 Table: PCR primers used in this study for *E. coli* detection and virulence genes**

| **Target gene** | **Primer name** | **Nucleotide sequence (5‘–3')** | **Annealing temp (°C)** | **Product size (bp)** | **References** |
| --- | --- | --- | --- | --- | --- |
| *uidA* | uidA-F | AAAACGGCAAGAAAAAGCAG | 56 | 147 | Bej et al. [1] |
|  | uidA-R | ACGCGTGGTTAACAGTCTTGCG |  |  |  |
| *St* | ST-F | GCTAAACCAGTAG AGGTCTTCAAAA | 57 | 147 | Nguyen et al. [2] |
|  | ST-R | CCCGGTACAGAGCAGGATTACAACA |  |  |  |
| *lt* | LT-F | CACACGGAGCTCCTCAGT C | 57 | 508 | Talukdar et al. [3] |
|  | LT-R | CCC CCA GCC TAG CTT AGT TT |  |  |  |
| *bfpA* | bfpA-F | GGAAGTCAAATTCATGGGGG | 57 | 300 | Talukdar et al. [3] |
|  | bfpA-R | GGAATCAGACGCAGACTGGT |  |  |  |
| *eae* | eae-F | CCCGAATTCGGCACAAGCATAAGC | 57 | 881 | Oswald et al. [4] |
|  | eae-R | CCCGGATCCGTCTCGCCAGTATTCG |  |  |  |
| *aat* | Pcvd432-F | CTGGCGAAAGACTGTATCAT | 57 | 650 | Mohamed et al. [5] |
|  | pcvd432-R | CAATGTATAGAAATCCGCTGTT |  |  |  |
| *aaiC* | AAIC-F | ATTGTCCTCAGG CATTTCAC | 57 | 215 | Talukdar et al. [3] |
|  | AAIC-R | CGACACCCCTGATAAACAA |  |  |  |
| *stx1* | stx1F | CACAATCAGGCGTCGCCAGCGCACTTGCT | 58 | 606 | Islam et al. [6] |
|  | stx1R | TGTTGCAGGGATCAGTGGTACGGGGATGC |  |  |  |
| *stx2* | stx2F | CCACATCGGTGTCTGTTATTAACCACACC | 58 | 372 | Heuvelink et al. [7] |
|  | stx2R | GCAGAACTGCTCTGGATGCATCTCTGGTC |  |  |  |
| *iaa* | ial upper | CTGGATGGTATGGTGAGG | 57 | 320 | Frankel et al. [8] |
|  | ial lower | GGAGGCCAACAATTATTTCC |  |  |  |
| *ipaH* | Shig-1 | TGGAAAAACTCAGTGCCTCT | 57 | 424 | Lüscher and Altwegg [9] |
|  | Shig-2 | CCAGTCCGTAAATTCATTCT |  |  |  |


**S2a Table. Phenotypic profile of *E. coli* isolates**

| **Sample**  **source** | **Isolate**  **Code** | **Isolate source** | **Resistance phenotype** | **NRA** | **NOA** | **MARI** |
| --- | --- | --- | --- | --- | --- | --- |
| Restaurant | EYJ012 | Jollof rice | AMP^R^, CTX^R^, CAZ^R^ | 3 | 2 | 0.19 |
| Street | OKA017 | Okazi soup | AZM^R^, AZT^R^, TET^R^, CHL^R^, CIP^R^, NAL^R^, CAZ^R^, CTX^R^, AMP^R^, AMS^R^, AMC^R^ | 11 | 8 | 0.69 |
| Cafeteria | OGS018 | Oil and garri soup | AMP^R^, CTX^R^ | 2 | 2 | 0.13 |
| Cafeteria | COR021 | Coconut rice | AMP^R^, CTX^R^, CAZ^R^, AZM^R^ | 4 | 3 | 0.25 |
| Street | VGS023 | Vegetable soup | AZM^R^, AZT^R^, TET^R^, CHL^R^, CIP^R^, NAL^R^, CAZ^R^, CTX^R^, AMP^R^, AMS^R^, AMC^R^, STX^R^, STR^R^ | 13 | 10 | 0.81 |
| Street | DIP024 | Yam porridge | AZM^R^, AZT^R^, TET^R^, CHL^R^, CIP^R^, CAZ^R^, CTX^R^, AMP^R^, AMS^R^ | 9 | 8 | 0.56 |
| Street | MES028 | Melon soup | AMS^R^, AMC^R^, AMP^R^, CHL^R^, STX^R^, TET^R^, AZT^R^, AZM^R^ | 8 | 7 | 0.5 |
| Street | BLS030 | Bitter leave soup | AZM^R^, AZT^R^, TET^R^, CHL^R^, CIP^R^, NAL^R^, CAZ^R^, CTX^R^, AMP^R^, AMS^R^, AMC^R^ | 11 | 8 | 0.69 |
| Street | COS039 | Cocoyam soup | AZM^R^, AZT^R^, TET^R^, CHL^R^, CIP^R^, CAZ^R^, CTX^R^, AMP^R^, AMS^R^ | 9 | 8 | 0.56 |
| Street | FRR046 | Fried rice | AMP^R^, NAL^R^, CIP^R^, CHL^R^, STX^R^, TET^R^, AZM^R^ | 7 | 6 | 0.44 |
| Street | OKA057 | Okazi soup | AZM^R^, AZT^R^, TET^R^, CHL^R^, CIP^R^, NAL^R^, CAZ^R^, CTX^R^, AMP^R^, AMS^R^, AMC^R^, STX^R^, STR^R^ | 13 | 10 | 0.81 |
| Cafeteria | OGS061 | Oil and garri soup | AMS^R^, AMC^R^, AMP^R^ | 3 | 2 | 0.19 |
| Street | VGS068 | Vegetable soup | AZM^R^, AZT^R^, TET^R^, CHL^R^, CIP^R^, NAL^R^, CAZ^R^, CTX^R^, AMP^R^, AMS^R^, AMC^R,^ NIT^R^ | 12 | 9 | 0.75 |
| Street | FRB071 | Fried beans | AMP^R^, NAL^R^, CIP^R^, CHL^R^, STX^R^, TET^R^, AZM^R^ | 7 | 6 | 0.44 |
| Cafeteria | JOR072 | Jollof rice | AMP^R^, NAL^R^, CIP^R^, CHL^R^, STX^R^, AZM^R^ | 6 | 5 | 0.38 |
| Street | PES080 | Pepper soup | AZM^R^, AZT^R^, TET^R^, CHL^R^, CIP^R^, NAL^R^, CAZ^R^, CTX^R^, AMP^R^, AMS^R^, AMC^R^, STX^R^, NIT^R^, STR^R^, GEN^R^ | 15 | 11 | 0.94 |
| Street | MES084 | Melon soup | AZM^R^, AMP^R^, TET^R^, CIP^R^, CHL^R^, STX^R^ | 6 | 6 | 0.44 |
| Restaurant | FRR087 | Fried rice | AMP^R^, CTX^R^ | 2 | 2 | 0.13 |
| Street | OKA091 | Okazi soup | AZM^R^, AZT^R^, TET^R^, CHL^R^, CIP^R^, NAL^R^, CAZ^R^, CTX^R^, AMP^R^, AMS^R^, AMC^R,^ NIT^R^ | 12 | 9 | 0.75 |
| Street | OGS097 | Oil and garri soup | AZM^R^, AMP^R^, TET^R^, CHL^R^, AZT^R^ | 5 | 5 | 0.38 |
| Street | VGS101 | Vegetable soup | AZM^R^, AZT^R^, TET^R^, CHL^R^, CIP^R^, NAL^R^, CAZ^R^, CTX^R^, AMP^R^, AMS^R^, AMC^R^, STX^R^, STR^R^ | 13 | 10 | 0.81 |
| Cafeteria | JOR102 | Jollof rice | AMP^R^, NAL^R^, CIP^R^, AZM^R^ | 4 | 3 | 0.25 |
| Street | OKS106 | Okro soup | AZM^R^, AZT^R^, TET^R^, CHL^R^, CIP^R^, NAL^R^, CAZ^R^, CTX^R^, AMP^R^, AMS^R^, AMC^R^, STX^R^, STR^R^ | 13 | 10 | 0.81 |
| Street | COS107 | Cocoyam soup | AZM^R^, AMP^R^, TET^R^, CHL^R^, STX^R^ | 5 | 5 | 0.38 |
| Street | OGS122 | Oil and garri soup | AMP^R^, NAL^R^, CIP^R^ | 3 | 2 | 0.19 |
| Street | MES123 | Melon soup | AMP^R^, NAL^R^, CIP^R^, CHL^R^, TET^R^, AZT^R^, AZM^R^ | 7 | 6 | 0.44 |
| Street | BAS134 | Banga soup | AMS^R^, AMC^R^, AMP^R^ | 3 | 2 | 0.19 |
| Street | POB142 | Porage beans | AMP^R^, CTX^R^ | 2 | 2 | 0.13 |
| Street | VGS148 | Vegetable soup | AZM^R^, AZT^R^, TET^R^, CHL^R^, CIP^R^, NAL^R^, CAZ^R^, CTX^R^, AMP^R^, AMS^R^, AMC^R^, STX^R^, STR^R^ | 13 | 10 | 0.81 |
| Street | OGS151 | Oil and garri soup | AMS^R^, AMC^R^, AMP^R^, AZM^R^ | 4 | 3 | 0.25 |
| Street | MES157 | Melon soup | AZM^R^, AZT^R^, TET^R^, CHL^R^, CIP^R^, CAZ^R^, CTX^R^, AMP^R^, AMS^R^ | 9 | 8 | 0.56 |
| Street | BAS162 | Banga soup | AMP^R^, NAL^R^, CIP^R^, CHL^R^, TET^R^, AZT^R^, AZM^R^ | 7 | 6 | 0.44 |
| Street | MSP171 | . Plantain porridge | AZM^R^, AZT^R^, TET^R^, CHL^R^, CIP^R^, CAZ^R^, CTX^R^, AMP^R^, AMS^R^ | 9 | 8 | 0.56 |
| Cafeteria | COR181 | Coconut rice | AMP^R^, CTX^R^, CAZ^R^ | 3 | 2 | 0.19 |
| Street | MES192 | Melon soup | AMS^R^, AMC^R^, AMP^R^, AZM^R^ | 4 | 3 | 0.25 |
| Street | COS199 | Cocoyam soup | AZM^R^, AZT^R^, TET^R^, CHL^R^, CIP^R^, CAZ^R^, CTX^R^, AMP^R^, AMS^R^ | 9 | 8 | 0.56 |
| Street | OKA206 | Okazi soup | AZM^R^, AZT^R^, TET^R^, CHL^R^, CIP^R^, NAL^R^, CAZ^R^, CTX^R^, AMP^R^, AMS^R^, AMC^R^ | 11 | 8 | 0.69 |
| Street | VGS210 | Vegetable soup | AZM^R^, AZT^R^, TET^R^, CHL^R^, CIP^R^, NAL^R^, CAZ^R^, CTX^R^, AMP^R^, AMS^R^, AMC^R^, STX^R^, STR^R^ | 13 | 10 | 0.81 |
| Cafeteria | DSP211 | Yam porridge | AMP^R^, GEN^R^, STR^R^ | 3 | 2 | 0.19 |
| Street | BLS213 | Bitter leave soup | AZM^R^, AZT^R^, TET^R^, CHL^R^, CIP^R^, NAL^R^, CAZ^R^, CTX^R^, AMP^R^, AMS^R^, AMC^R^, STX^R^, STR^R^ | 13 | 10 | 0.81 |
| Street | OKS214 | Okro soup | AZM^R^, AZT^R^, TET^R^, CHL^R^, CIP^R^, NAL^R^, CAZ^R^, CTX^R^, AMP^R^, AMS^R^, AMC^R^ | 11 | 8 | 0.69 |
| Cafeteria | POB215 | Porage beans | AMC^R^, AMP^R^, CHL^R^ | 3 | 3 | 0.19 |
| Restaurant | MES219 | Melon soup | AMS^R^, AMC^R^, AMP^R^ | 3 | 2 | 0.19 |
| Street | COS221 | Cocoyam soup | AZM^R^, AZT^R^, TET^R^, CHL^R^, CIP^R^, CAZ^R^, CTX^R^, AMP^R^, AMS^R^ | 9 | 8 | 0.56 |
| Restaurant | JOR223 | Jollof rice | AMP^R^, GEN^R^, STR^R^ | 3 | 2 | 0.19 |
| Street | VGS224 | Vegetable soup | AZM^R^, AZT^R^, TET^R^, CHL^R^, CIP^R^, NAL^R^, CAZ^R^, CTX^R^, AMP^R^, AMS^R^, AMC^R^ | 11 | 8 | 0.69 |
| Street | OKA229 | Okazi soup | AZM^R^, AZT^R^, TET^R^, CHL^R^, CIP^R^, NAL^R^, CAZ^R^, CTX^R^, AMP^R^, AMS^R^, AMC^R^ | 11 | 8 | 0.69 |
| Street | DSP232 | Yam porridge | AMP^R^, GEN^R^, NAL^R^, CIP^R^, CHL^R^, STX^R^, TET^R^, AZM^R^ | 8 | 6 | 0.5 |
| Cafeteria | BAS243 | Banga soup | AMP^R^, GEN^R^, STR^R^, CHL^R^, AZT^R^, AZM^R^ | 6 | 5 | 0.38 |
| Cafeteria | POB247 | Porage beans | AMS^R^, AMC^R^, AMP^R^ | 3 | 2 | 0.19 |
| Cafeteria | MES250 | Melon soup | AMS^R^, AMC^R^, AMP^R^, AZM^R^ | 4 | 3 | 0.25 |
| Street | COS257 | Cocoyam soup | AZM^R^, AZT^R^, TET^R^, CHL^R^, CIP^R^, CAZ^R^, CTX^R^, AMP^R^, AMS^R^ | 9 | 8 | 0.56 |
| Street | OKS268 | Okro soup | AZM^R^, AZT^R^, TET^R^, CHL^R^, CIP^R^, NAL^R^, CAZ^R^, CTX^R^, AMP^R^, AMS^R^, AMC^R^, STX^R^, STR^R^ | 13 | 10 | 0.81 |
| Restaurant | COR271 | Coconut rice | AMP^R^, GEN^R^, STR^R^ | 3 | 2 | 0.19 |
| Street | OKS272 | Okro soup | AZM^R^, AZT^R^, TET^R^, CHL^R^, CIP^R^, CAZ^R^, CTX^R^, AMP^R^, AMS^R^ | 9 | 8 | 0.56 |
| Street | MSP273 | Plantain porridge | AMP^R^, GEN^R^, NAL^R^, CIP^R^, CHL^R^, STX^R^, TET^R^, AZT^R^, AZM^R^, | 9 | 7 | 0.56 |
| Street | VGS276 | Vegetable soup | AZM^R^, AZT^R^, TET^R^, CHL^R^, CIP^R^, NAL^R^, CAZ^R^, CTX^R^, AMP^R^, AMS^R^, AMC^R^ | 11 | 8 | 0.69 |
| Restaurant | FRB279 | Fried beans | AMP^R^, GEN^R^ | 2 | 2 | 0.13 |
| Street | OKA280 | Okazi soup | AZM^R^, AZT^R^, TET^R^, CHL^R^, CIP^R^, NAL^R^, CAZ^R^, CTX^R^, AMP^R^, AMS^R^, AMC^R^, STX^R^, STR^R^ | 13 | 10 | 0.81 |
| Street | BAR281 | Banga rice | AMP^R^, GEN^R^, STR^R^, CHL^R^, AZT^R^, AZM^R^ | 6 | 5 | 0.38 |
| Street | COS282 | Cocoyam soup | AZM^R^, AMP^R^, TET^R^, CHL^R^, AZT^R^, AMS^R^ | 6 | 6 | 0.44 |
| Street | VGS296 | Vegetable soup | AZM^R^, AZT^R^, TET^R^, CHL^R^, CIP^R^, NAL^R^, CAZ^R^, CTX^R^, AMP^R^, AMS^R^, AMC^R^, STX^R^, STR^R^ | 13 | 10 | 0.81 |

**Legend**: NRA: No resistant antibiotics; NOAC: No of antimicrobial class

**S2b Table. Phenotypic and genotypic characterization of *E. coli* isolates**

|  |  |  | **Virulence determinants** | | | | | | | | | | **Extracellular virulence factors** | | | | | | | |
| --- | --- | --- | --- | --- | --- | --- | --- | --- | --- | --- | --- | --- | --- | --- | --- | --- | --- | --- | --- | --- |
| **Sample**  **source** | **Isolate**  **Code** | **Isolate source** | ***st*** | ***lt*** | ***bfp*** | ***eae*** | ***aat*** | ***AaiC*** | ***stx1*** | ***stx2*** | ***ipaH*** | ***ial*** | **PA** | **CUF** | **CEF** | **LYD** | **ORD** | **AEH** | **BGA** | **BEL** |
| Restaurant | EYJ012 | Jollof rice |  |  |  |  |  |  |  |  |  | + |  |  |  | + | + |  | + | + |
| Street | OKA017 | Okazi soup |  |  | + | + |  |  |  |  |  |  |  |  |  | + |  |  | + | + |
| Cafeteria | OGS018 | Oil and garri soup |  |  |  |  | + | + |  |  |  |  |  |  |  | + |  |  | + | + |
| Cafeteria | COR021 | Coconut rice |  |  |  |  |  |  |  |  | + | + |  | + |  | + | + |  | + | + |
| Street | VGS023 | Vegetable soup |  |  | + | + |  |  |  |  |  |  | + | + | + | + | + |  | + | + |
| Street | DIP024 | Yam porridge |  |  |  |  |  | + |  |  |  |  |  | + |  | + |  |  | + | + |
| Street | MES028 | Melon soup |  |  |  |  | + | + |  |  |  |  |  | + |  | + |  |  | + | + |
| Street | BLS030 | Bitter leave soup | + | + |  |  |  |  |  |  |  |  |  | + | + | + |  | + | + | + |
| Street | COS039 | Cocoyam soup |  |  | + | + |  |  |  |  |  |  |  | + |  | + |  |  | + | + |
| Street | FRR046 | Fried rice |  |  |  |  |  |  |  |  |  |  |  | + |  | + | + |  | + | + |
| Street | OKA057 | Okazi soup | + | + |  |  |  |  |  |  |  |  | + | + | + | + | + | + | + | + |
| Cafeteria | OGS061 | Oil and garri soup |  |  |  |  |  |  |  |  |  |  |  |  |  | + |  |  | + | + |
| Street | VGS068 | Vegetable soup | + | + |  |  |  |  |  |  |  |  |  | + | + | + |  | + | + | + |
| Street | FRB071 | Fried beans |  |  |  |  |  | + |  |  |  |  |  | + |  | + |  |  | + | + |
| Cafeteria | JOR072 | Jollof rice |  |  |  |  |  |  |  |  |  | + |  | + |  | + |  |  | + | + |
| Street | PES080 | Pepper soup | + | + |  |  |  |  |  |  |  |  |  | + | + | + |  | + | + | + |
| Street | MES084 | Melon soup |  | + |  |  |  |  |  |  |  |  |  | + |  | + | + | + | + | + |
| Restaurant | FRR087 | Fried rice |  |  |  |  |  |  |  |  |  |  |  |  |  | + |  |  | + | + |
| Street | OKA091 | Okazi soup |  |  | + | + |  |  |  |  |  |  | + | + | + | + | + | + | + | + |
| Street | OGS097 | Oil and garri soup |  |  | + | + |  |  |  |  |  |  |  | + |  | + |  | + | + | + |
| Street | VGS101 | Vegetable soup | + | + |  |  |  |  |  |  |  |  |  | + |  | + |  | + | + | + |
| Cafeteria | JOR102 | Jollof rice |  |  |  |  |  | + |  |  |  |  | + |  |  | + | + |  | + | + |
| Street | OKS106 | Okro soup |  |  | + | + |  |  |  |  |  |  | + | + | + | + | + |  | + | + |
| Street | COS107 | Cocoyam soup |  | + |  |  |  |  |  |  |  |  |  |  |  | + |  | + | + | + |
| Street | OGS122 | Oil and garri soup |  |  |  | + |  |  | + |  |  |  |  |  |  | + |  |  | + | + |
| Street | MES123 | Melon soup |  |  |  |  |  |  |  |  |  |  |  |  |  | + |  |  | + | + |
| Street | BAS134 | Banga soup |  |  |  |  |  |  |  |  |  |  | + |  |  | + | + |  | + | + |
| Street | POB142 | Porage beans |  |  |  |  | + | + |  |  |  |  |  |  |  | + |  |  | + | + |
| Street | VGS148 | Vegetable soup |  | + |  |  |  |  |  |  |  |  |  | + | + | + | + |  | + | + |
| Street | OGS151 | Oil and garri soup |  |  |  |  |  |  |  |  | + | + |  |  |  | + |  |  | + | + |
| Street | MES157 | Melon soup |  | + |  |  |  |  |  |  |  |  | + | + | + | + | + |  | + | + |
| Street | BAS162 | Banga soup |  |  | + | + |  |  |  |  |  | + |  |  |  | + |  |  | + | + |
| Street | MSP171 | Plantain porridge |  |  |  | + |  |  | + | + |  |  |  | + | + | + |  |  | + | + |
| Cafeteria | COR181 | Coconut rice |  |  |  |  |  |  |  |  |  |  |  |  |  | + |  |  | + | + |
| Street | MES192 | Melon soup |  |  | + | + |  |  |  |  |  |  |  |  |  | + |  |  | + | + |
| Street | COS199 | Cocoyam soup |  |  |  |  |  |  |  |  |  |  |  | + |  | + |  |  | + | + |
| Street | OKA206 | Okazi soup | + | + |  |  |  |  |  |  |  |  |  | + | + | + |  | + | + | + |
| Street | VGS210 | Vegetable soup |  | + |  |  |  |  |  |  |  |  | + | + | + | + | + |  | + | + |
| Cafeteria | DSP211 | Yam porridge |  |  | + | + |  |  |  |  |  |  |  |  |  | + | + |  | + | + |
| Street | BLS213 | Bitter leave soup |  | + |  |  |  |  |  |  |  |  |  | + | + | + |  |  | + | + |
| Street | OKS214 | Okro soup |  | + |  |  |  |  |  |  |  |  |  | + | + | + |  |  | + | + |
| Cafeteria | POB215 | Porage beans |  |  |  |  | + | + |  |  |  |  |  |  |  | + |  |  | + | + |
| Restaurant | MES219 | Melon soup |  |  | + | + |  |  |  |  |  |  |  |  |  | + |  |  | + | + |
| Street | COS221 | Cocoyam soup |  |  |  |  |  |  |  |  |  |  |  | + |  | + |  |  | + | + |
| Restaurant | JOR223 | Jollof rice |  |  |  |  |  | + |  |  |  |  |  |  |  | + |  |  | + | + |
| Street | VGS224 | Vegetable soup | + | + |  |  |  |  |  |  |  |  | + | + | + | + | + | + | + | + |
| Street | OKA229 | Okazi soup |  | + |  |  |  |  |  |  |  |  |  | + | + | + |  |  | + | + |
| Street | DSP232 | Yam porridge |  |  |  |  | + | + |  |  |  |  |  | + |  | + |  |  | + | + |
| Cafeteria | BAS243 | Banga soup |  |  |  |  |  |  |  |  |  |  |  | + | + | + |  |  | + | + |
| Cafeteria | POB247 | Porage beans |  |  |  |  | + | + |  |  |  |  |  |  |  | + |  |  | + | + |
| Cafeteria | MES250 | Melon soup |  |  |  |  |  |  |  |  |  |  |  |  |  | + |  |  | + | + |
| Street | COS257 | Cocoyam soup |  |  | + | + |  |  |  |  |  |  | + | + |  | + | + |  | + | + |
| Street | OKS268 | Okro soup | + | + |  |  |  |  |  |  |  |  |  | + | + | + |  | + | + | + |
| Restaurant | COR271 | Coconut rice |  |  |  |  |  | + |  |  |  |  |  |  |  | + |  |  | + | + |
| Street | OKS272 | Okro soup | + | + |  |  |  |  |  |  |  |  |  | + |  | + |  | + | + | + |
| Street | MSP273 | Plantain porridge |  |  |  | + |  |  |  |  |  |  |  | + |  | + |  |  | + | + |
| Street | VGS276 | Vegetable soup |  |  | + | + |  |  |  |  |  |  | + | + | + | + | + |  | + | + |
| Restaurant | FRB279 | Fried beans |  |  |  |  |  |  |  |  |  |  |  |  |  | + | + |  | + | + |
| Street | OKA280 | Okazi soup |  | + |  |  |  |  |  |  |  |  |  | + | + | + |  |  | + | + |
| Street | BAR281 | Banga rice |  |  |  |  |  |  |  |  |  |  |  | + |  | + |  |  | + | + |
| Street | COS282 | Cocoyam soup |  |  | + | + |  |  |  |  |  |  |  | + |  | + |  |  | + | + |
| Street | VGS296 | Vegetable soup |  | + |  |  |  |  |  |  |  |  | + | + | + | + | + | + | + | + |

**Legend**: PA: Protease activity, CUF: Curli formation, CEF: Cellulose formation, ORD: Ornithine decarboxylase, AEH: Aesculin hydrolysis, LYD: Lysine decarboxylase, BGA: Beta-galactosidase, BEL: Beta-lactamase

**S3a Table. Correlation of phenotypic characteristics of *E. coli***

|  | PA | CUF | CEF | ORD | AEH |
| --- | --- | --- | --- | --- | --- |
| PA | 1.000 |  |  |  |  |
| CUF | 0.207 | 1.000 |  |  |  |
| CEF | 0.426** | 0.550** | 1.000 |  |  |
| ORD | 0.737** | 0.148 | 0.263* | 1.000 |  |
| AEH | 0.126 | 0.335** | 0.347** | 0.059 | 1.000 |

**Legend**: RA: resistant antibiotics, SS: sample source, PA: protease activity, CUF: curli formation, CEF: cellulose formation, ORD: ornithine decarboxylase, AEH: aesculin hydrolysis

**S3b Table. Correlation matrix of phenotypic and genotypic characteristics of *E. coli***

|  | SS | No RA | MARI | *St* | *Lt* | *bfp* | *eae* | *aat* | *aaiC* | *stx1* | *stx2* | *ipaH* | *ial* |
| --- | --- | --- | --- | --- | --- | --- | --- | --- | --- | --- | --- | --- | --- |
| SS | 1 |  |  |  |  |  |  |  |  |  |  |  |  |
| No RA | 0.649** | 1.000 |  |  |  |  |  |  |  |  |  |  |  |
| MARI | 0.663** | 0.998** | 1.000 |  |  |  |  |  |  |  |  |  |  |
| *st* | 0.239 | 0.465** | 0.463** | 1.000 |  |  |  |  |  |  |  |  |  |
| *lt* | 0.377** | 0.634** | 0.647** | 0.619** | 1.000 |  |  |  |  |  |  |  |  |
| *bfp* | 0.111 | 0.072 | 0.084 | -0.217 | -0.342** | 1.000 |  |  |  |  |  |  |  |
| *eae* | 0.166 | 0.049 | 0.058 | -0.248 | -0.392** | 0.873** | 1.000 |  |  |  |  |  |  |
| *Aat* | -0.065 | -0.276* | -0.284* | -0.137 | -0.218 | -0.169 | -0.193 | 1.000 |  |  |  |  |  |
| *aaiC* | -0.253* | -0.345** | -0.356** | -0.195 | -0.309* | -0.239 | -0.274* | 0.705** | 1.000 |  |  |  |  |
| *stx1* | 0.104 | -0.076 | -0.081 | -0.077 | -0.121 | -0.094 | 0.310* | -0.060 | -0.085 | 1.000 |  |  |  |
| *stx2* | 0.073 | 0.046 | 0.042 | -0.054 | -0.085 | -0.066 | 0.217 | -0.042 | -0.059 | 0.701** | 1.000 |  |  |
| *ipaH* | -0.036 | -0.170 | -0.176 | -0.077 | -0.121 | -0.094 | -0.108 | -0.060 | -0.085 | -0.033 | -0.023 | 1.000 |  |
| *Ial* | -0.194 | -0.215 | -0.221 | -0.124 | -0.197 | -0.007 | -0.039 | -0.097 | -0.138 | -0.054 | -0.038 | 0.616** | 1.000 |
| PA | 0.216 | 0.343** | 0.336** | 0.027 | 0.117 | 0.249 | 0.178 | -0.160 | -0.121 | -0.089 | -0.063 | -0.089 | -0.145 |
| CUF | 0.586** | 0.758** | 0.767** | 0.324** | 0.438** | -0.015 | -0.005 | -0.200 | -0.255* | -0.049 | 0.098 | -0.049 | -0.140 |
| CEF | 0.354** | 0.756** | 0.751** | 0.380** | 0.633** | -0.034 | -0.033 | -0.234 | -0.332** | 0.062 | 0.179 | -0.131 | -0.212 |
| ORD | 0.003 | 0.156 | 0.154 | -0.066 | 0.089 | 0.173 | 0.088 | -0.218 | -0.217 | -0.121 | -0.085 | 0.077 | 0.060 |
| AEH | 0.307* | 0.421** | 0.448** | 0.762** | 0.645** | -0.089 | -0.142 | -0.177 | -0.251* | -0.099 | -0.069 | -0.099 | -0.160 |

**Legend**: RA: resistant antibiotics, SS: Sample source, PA: Protease activity, CUF: Curli formation, CEF: Cellulose formation, ORD: Ornithine decarboxylase, AEH: Aesculin hydrolysis

**References**

1. Bej AK, DiCesare JL, Haff L, Atlas RM. Detection of *Escherichia coli* and *Shigella* spp. in water by using the polymerase chain reaction and gene probes for *uid*. Appl Environ Microbiol. 1991; 57: 1013–1017.
2. Nguyen TV, Le Van P, Le Huy C, Gia KN, Weintraub A. Detection and characterization of diarrheagenic *Escherichia coli* from young children in Hanoi, Vietnam. J Clin Microbiol. 2005; 43: 755–760.
3. Talukdar PK, Rahman M, Rahman M, Nabi A, Islam Z, Hoque MM. Antimicrobial resistance, virulence factors and genetic diversity of *Escherichia coli* isolates from household water supply in Dhaka, Bangladesh. PLoS ONE. 2013; 8: e61090.
4. Oswald E, Schmidt H, Morabito S, Karch H, Marches O, Caprioli A. Typing of intimin genes in human and animal enterohemorrhagic and enteropathogenic *Escherichia coli*: characterization of a new intimin variant. Infect Immun. 2000; 68: 64–71.
5. Mohamed JA, DuPont HL, Jiang ZD, Flores J, Carlin LG, BelkindGerson J. A single-nucleotide polymorphism in the gene encoding osteoprotegerin, an anti-inflammatory protein produced in response to infection with diarrheagenic *Escherichia coli*, is associated with an increased risk of nonsecretory bacterial diarrhea in North Ame. J Infect Dis. 2009; 199: 477–485.
6. Islam MA, Heuvelink AE, De Boer E, Sturm PD, Beumer RR, Zwietering MH. Shiga toxin-producing *Escherichia coli* isolated from patients with diarrhoea in Bangladesh. J Med Microbiol. 2007; 56: 380–385.
7. Heuvelink AE, Van de Kar N, Meis J, Monnens LAH, Melchers WJG. Characterization of verocytotoxin-producing *Escherichia coli* O157 isolates from patients with haemolytic uraemic syndrome in Western Europe. Epidemiol Infect. 1995; 115: 1–3.
8. Frankel G, Giron JA, Valmassoi J, Schoolnik GK. Multi-gene amplification: simultaneous detection of three virulence genes in diarrhoeal stool. Mol Microbiol. 1989; 3: 1729–1734.
9. Lüscher D, Altwegg M. Detection of shigellae, enteroinvasive and enterotoxigenic *Escherichia coli* using the polymerase chain reaction (PCR) in patients returning from tropical countries. Mol Cell Probes. 1994; 8: 285–90.
